# Coexistence of ulcerative colitis and neurofibromatosis type 1: a case report and literature review

**DOI:** 10.3389/fmed.2025.1723026

**Published:** 2025-11-19

**Authors:** Xiuqiang Huang, Shuhua Lyu, Jin Zhong, Yiming Li, Luyao Zhao, Manyi Sun

**Affiliations:** 1 Department of Gastroenterology, Tianjin Union Medical Center, The First Affiliated Hospital of Nankai University, Tianjin, China; 2 Department of Pathology, Tianjin Union Medical Center, The First Affiliated Hospital of Nankai University, Tianjin, China; 3 Department of Radiology, Tianjin Union Medical Center, The First Affiliated Hospital of Nankai University, Tianjin, China

**Keywords:** UC, coexistence, polyps, subcutaneous nodules, NF1

## Abstract

**Background:**

Neurofibromatosis type 1 (NF1) is an autosomal dominant genetic disorder resulting from mutations in the NF1 gene, while ulcerative colitis (UC) is a chronic inflammatory disease of the intestines with an unclear etiology. Herein, we report a case involving an elderly male patient with both UC and NF1.

**Case presentation:**

The patient experienced chronic diarrhea and mucopurulent bloody stools, leading to a confirmed diagnosis of UC through colonoscopy and histopathological examination. Treatment with corticosteroids, vedolizumab, and five-flavor sophora flavescens enteric-coated capsules (SFEC), a traditional Chinese medicine, successfully achieved remission. During the physical examination, the patient exhibited axillary café-au-lait spots, scoliosis, and multiple subcutaneous nodules on the head, trunk, and limbs. Imaging and histopathological analysis identified these nodules as neurofibromas, fulfilling the diagnostic criteria for NF1. Key features of this case include the steroid-dependent nature of the UC, which significantly improved with the combination therapy of vedolizumab and SFEC, as well as the presence of neurofibromas affecting the intestines and greater omentum. Furthermore, colonic ulcers and polypoid lesions may have been linked to the neurofibromas, while histopathological findings in both skin and intestinal tissues indicated the involvement of mast cells. This case illustrates the intricate relationship between UC and NF1, supporting a common inflammatory mechanism that may involve mast cells.

**Conclusion:**

This case highlights the importance of clinicians being aware of the rare coexistence of UC and NF1 to ensure accurate diagnosis and appropriate management.

## Introduction

Neurofibromatosis type 1 (NF1) is an autosomal dominant disorder caused by mutations in the NF1 gene on chromosome 17 (17q11.2) (1–3). The core pathological mechanism involves loss of function of neurofibromin, leading to dysregulated RAS signaling (1–3). NF1 displays a broad spectrum of clinical features, including café-au-lait spots, axillary/inguinal freckling, and neurofibromas, with potential involvement of the nervous, skeletal, and cardiovascular systems, illustrating considerable phenotypic heterogeneity (1, 4). Advances in molecular research have expanded diagnostic approaches from clinical criteria to genetic testing and imaging, though multisystem complexity continues to pose significant management challenges (3, 5). Epidemiologically, NF1 has an estimated prevalence of 1/2000–1/5000 (3), and diagnosis integrates clinical evaluation (e.g., NIH 1988 criteria) and genetic analysis (1, 4, 5).

Ulcerative colitis (UC) is a chronic inflammatory bowel disease of elusive etiology, associated with genetic susceptibility, epithelial barrier defects, and immune–microbial dysregulation (6, 7). Typical presentation includes bloody diarrhea with mucus, and diagnosis requires endoscopic evidence of continuous mucosal inflammation alongside characteristic histopathological changes such as crypt architectural distortion (5, 8). Although biologic therapies (e.g., anti-TNFα antibodies) have improved outcomes, 10%–20% of patients eventually require proctocolectomy, and long-term remission rates remain below 40% (6, 8). Extraintestinal manifestations of UC (e.g., cutaneous or articular involvement) may obscure diagnosis and complicate clinical management (8).

The co-occurrence of NF1 and UC is exceedingly rare, with potential mechanistic links involving shared genetic susceptibility or immune dysregulation. NF1-related gastrointestinal neurofibromas may manifest as wall thickening or polyploid lesions, while the proinflammatory milieu in UC, characterized by cytokines such as TNF-α and IL-6, could theoretically influence neurofibroma pathogenesis (8, 9). Furthermore, mast cell infiltration, a histopathological feature observed in both UC and NF1-associated neurofibromas, likely contributes to disease progression through histamine and cytokine release, which are implicated in intestinal inflammation and fibrosis (8–11). We report the case of an elderly male with steroid-dependent UC and NF1-associated neurofibromas involving the intestine and omentum, accompanied by histologically confirmed mast cell infiltration. This case report corroborates emerging evidence that mast cell activation may represent an inflammatory mechanism involved in both conditions.

## Patient information

### General information

The patient is a 61-year-old man with a 5-year history of intermittent diarrhea and mucopurulent bloody stools. He initially presented 5 years prior to admission with diarrhea without an identifiable trigger, experiencing > 20 bowel movements per day accompanied by mucopurulent bloody stool. Colonoscopy performed at an external hospital confirmed ulcerative colitis (pancolitis). Initial treatment with mesalamine and corticosteroids led to symptomatic improvement, reducing bowel movements to 4–5 per day and resolving mucopurulent bloody stool. However, symptoms recurred following corticosteroid taper, leading to the initiation of vedolizumab in July 2022. From July 2022 to January 2025, he received regular vedolizumab infusions (according to the standard induction regimen at weeks 0, 2, and 6, followed by maintenance dosing every 8 weeks), which sustained clinical remission (1–3 bowel movements per day, occasional hematochezia) until he self-discontinued the medication in January 2025.

A clinical relapse occurred in May 2025, characterized by > 10 bowel movements per day and mucopurulent bloody stools. The patient was initially evaluated in our hospital’s outpatient clinic and subsequently admitted for the first time in June 2025 for further management.

### Physical examination

**FIGURE 1:**
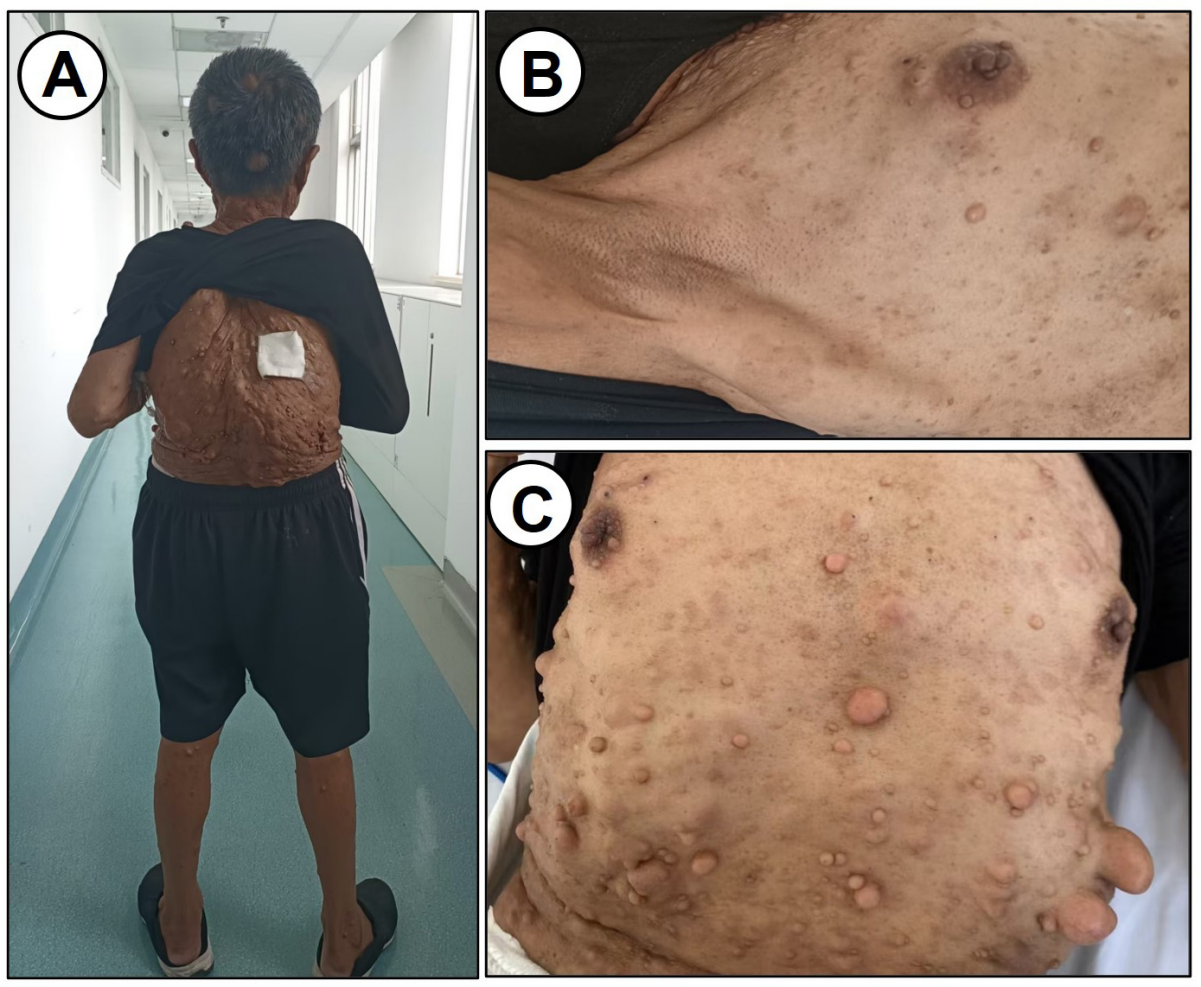
Physical examination data. **(A)** Characteristics of the back. **(B,C)** Subcutaneous nodules on the chest and abdomen are shown.

On physical examination, the patient was afebrile (36.5 °C) with a pulse of 76 bpm, respiratory rate of 16 breaths/min, and blood pressure of 125/80 mmHg. Patient’s Height was 142 cm. The patient was well-developed and moderately nourished. The patient also had short stature and scoliosis. Multiple café-au-lait spots were observed in the axillary region; the largest measuring approximately 2.5 × 1.0 cm. No freckling was observed. Numerous subcutaneous nodules, ranging from 1 to 3 cm, were distributed over the head, face, trunk, and limbs (Figure 1).

### Laboratory tests

Laboratory investigations showed a white blood cell count of 7.3 × 10^9^/L with 76.6% neutrophils, hemoglobin of 109 g/L, and platelets of 311 × 10^9/L^/L. Stool analysis revealed 4–6 white blood cells per HPF, 6–8 red blood cells per HPF, and positive occult blood. Stool culture and amoebic smear were negative. Clostridium difficile antigen testing was positive, but the toxin assay was negative. Blood CMV-DNA and EBV-DNA were undetectable. T-SPOT.TB results showed 5 sfc/2.5 × 10^5^ peripheral blood mononuclear cells for tuberculosis antigen A (TBA) and 7 sfc/2.5 × 10^5^ for tuberculosis antigen B (TBB). Biochemistry indicated albumin 34.1 g/L, C-reactive protein 10.10 mg/L, and erythrocyte sedimentation rate 48 mm/h. Serology was positive for pANCA at 1:10 titer and ANA at 1:100. IgM, IgA, IgG, C3, and C4 levels were within normal limits.

### Colonoscopy

**FIGURE 2:**
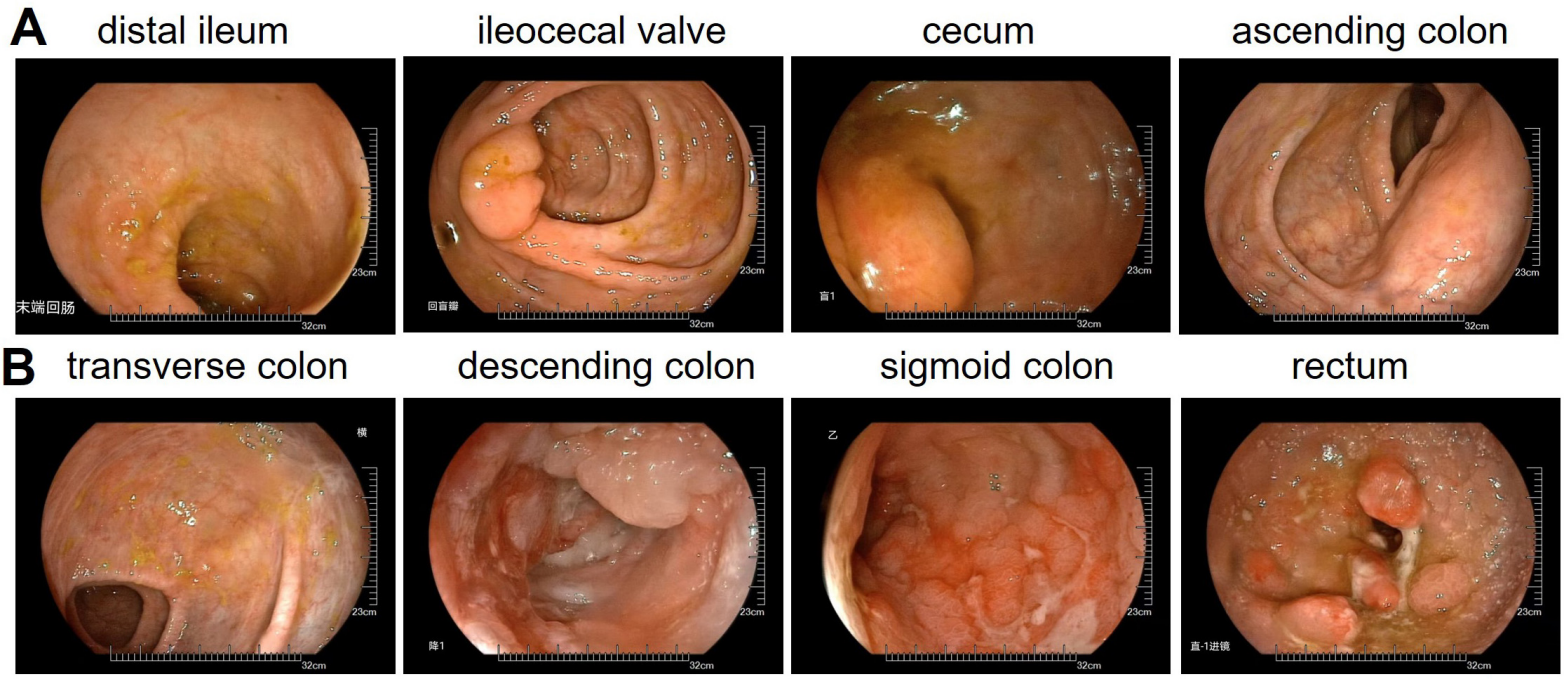
Colonoscopy examination data. **(A)** Distal ileum, ileocecal valve, cecum, ascending colon. **(B)** Transverse colon, descending colon, sigmoid colon, rectum.

Colonoscopy (Figure 2) identified a 1.8 cm elevated lesion in the cecum with mild surface erythema. The ascending colon appeared normal with preserved haustra and peristalsis. Scarring was observed in the transverse colon. Multiple mucosal erosions, ulcers, and polyps were present in the descending colon, sigmoid colon, and rectum. Biopsies from the descending colon, rectum, and rectal polyps were consistent with ulcerative colitis and associated inflammatory polyps. These findings support the diagnosis of ulcerative colitis.

### Histopathological examination

Histopathological evaluation of subcutaneous nodules revealed characteristic features of neurofibromas on hematoxylin and eosin (HE) staining (Supplementary Figure 1A). Immunohistochemistry was positive for S100 (Supplementary Figure 1B), confirming neural origin. Scattered CD117 positivity (Supplementary Figure 1C) suggested mast cell involvement. These findings, together with clinical features, confirm the diagnosis of NF1. This diagnosis fulfills the 1997 NIH diagnostic criteria for NF1.

**FIGURE 3:**
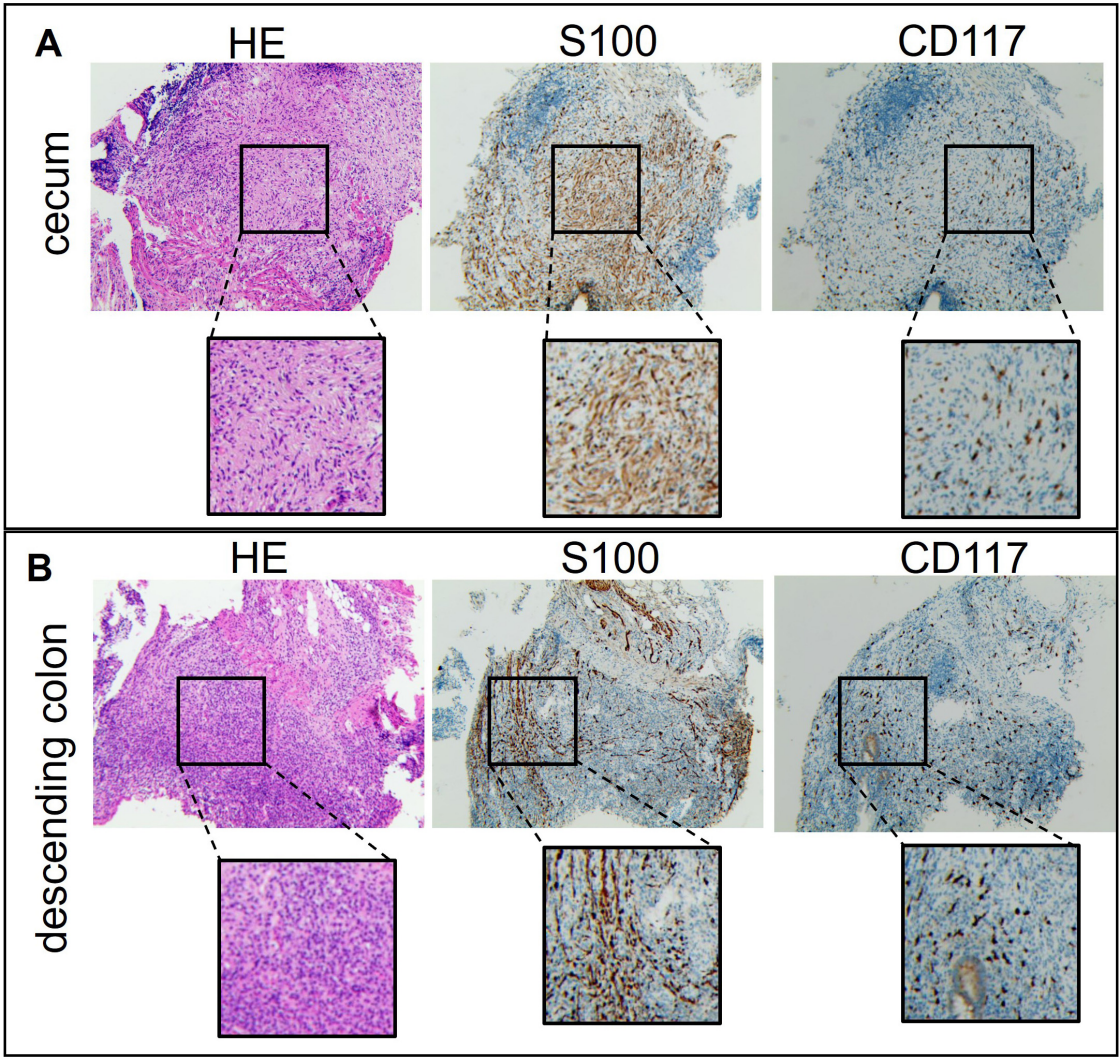
Colonoscopy examination data of the patient’s intestinal tissue. **(A)** Cecum. **(B)** Descending colon.

Examination of intestinal tissue from the cecum showed abnormal architecture on H&E staining (Figure 3A). S100 positivity supported the presence of neurofibroma, and scattered CD117 staining indicated mast cell participation. The lesion was composed of a neurofibroma with focal chronic inflammation. Sections from the descending colon and rectum (Figure 3B) demonstrated features typical of ulcerative colitis, including acute and chronic inflammation, crypt abscesses, and crypt branching and distortion. Focal S100 and CD117 positivity raised the possibility of local neurofibromatous involvement. These findings confirm intestinal neurofibroma and suggest a potential pathogenic role of mast cells in disease pathology.

### Imaging studies

**FIGURE 4:**
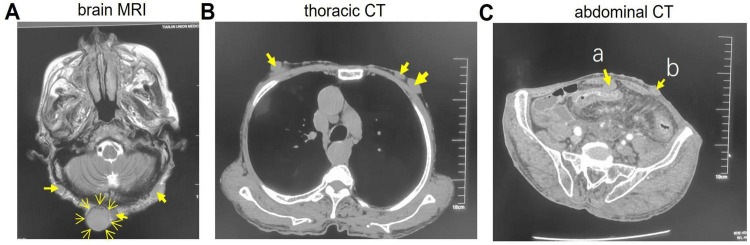
Imaging studies data. **(A)** Brain magnetic resonance imaging (MRI). **(B)** Thoracic CT. **(C)** Abdominal CT. The arrows indicate nodules. a, Omentum nodule; b, Abdominal nodule.

Brain magnetic resonance imaging (MRI) (Figure 4A) showed multiple punctate and patchy lesions with long T1 and T2 signals in the pons, basal ganglia, and thalamus, some isointense to cerebrospinal fluid, suggestive of lacunar infarcts. Multiple diffuse nodules were seen in the scalp and subcutaneous neck tissue (arrows), the largest in the occipital region (∼2.6 cm), with well-defined margins and long T1/slightly long T2 signals, possibly related to neurofibromatosis.

Thoracic CT (Figure 4B) revealed multiple subcutaneous nodules in the chest wall and back (arrows). Abdominal CT (Figure 4C) demonstrated diffuse circumferential wall thickening in the descending colon, sigmoid colon, and rectum, with loss of haustral folds, consistent with ulcerative colitis. Multiple nodules were also present in the omentum (arrow a), abdominal (arrow b) and pelvic walls, lumbar region, buttocks, and perineal subcutaneous tissue. Enlarged lymph nodes were noted in the para-aortic and superior rectal artery regions, possibly indicating inflammation or lymphadenopathy.

### Therapeutic process

The final diagnosis was UC co-occurring with NF1. During hospitalization, the patient was treated with mesalazine, antibiotics, and SFEC. At this admission, the patient received mesalazine and SFEC without vedolizumab. Given that the patient had previously shown symptomatic and colonoscopic improvement with vedolizumab therapy, and considering that the current relapse was associated with the patient’s self-discontinuation of vedolizumab, we recommended reinitiating vedolizumab to induce remission. Following treatment, the patient’s diarrhea resolved. At discharge, the frequency of bowel movements was 1–2 times per day, without mucous or bloody stool. Both the rapid C-reactive protein (CRP) test and erythrocyte sedimentation rate (ESR) returned to normal levels. Due to the short duration of treatment, follow-up abdominal CT and colonoscopy were not performed, as there was insufficient time to observe significant changes.

## Discussion

The co-occurrence of UC and NF1 is exceedingly rare (10, 12). Additionally, one case of Crohn’s disease with NF1 (13) and one case of NF1 with inflammatory bowel disease and primary sclerosing cholangitis (14) have been reported. The present case of an elderly male with UC and NF1 further broadens the reported age range, indicating that the comorbidity may occur across all ages, though the total number of published cases remains below 20 (9–15). Since the initial report in 1989, cases have been described in all age groups—from infancy to old age—without clear sex predilection, involving both males and females (11, 12, 14, 16).

Clinically, these cases combine intestinal inflammation from UC (e.g., bloody diarrhea, abdominal pain) with classic NF1 features (café-au-lait spots, neurofibromas, skeletal abnormalities). Notably, 30%–60% of NF1 patients may develop gastrointestinal stromal tumors (GIST), whose symptoms (e.g., bleeding) overlap with those of UC. Gastrointestinal manifestations in NF1 often stem from neurofibromas (10%–25%) and include bleeding, obstruction, or pain (9, 16), mimicking inflammatory bowel disease. Several reported patients presented with bloody stools and were initially misdiagnosed with NF1-related tumors before colonoscopy and biopsy confirmed UC (10–12, 16). The current case also exhibited mucopurulent bloody stools, requiring endoscopic and histopathological differentiation.

From a pathogenetic perspective, NF1 arises from autosomal dominant mutations in the NF1, leading to loss of neurofibromin and dysregulated cell growth and differentiation (1–3, 12). Though no specific recurrent mutations have been identified, these alterations disrupt cellular homeostasis, contributing to the condition’s hallmark features. UC, similarly, has a genetic component, with susceptibility influenced by variations in multiple genes. The co-occurrence of UC and NF1 may reflect shared genetic susceptibility, wherein certain variants predispose individuals to both diseases. Supporting this, NF1 exhibits high phenotypic variability—even among carriers of identical NF1 mutations—potentially encompassing immune dysregulation (3). Familial clustering of inflammatory bowel disease, including a reported case of CD in a family with NF1 (13), further underscores the possibility of overlapping genetic risk factors between these conditions.

Mast cells appear to play a role in both diseases. In NF1, they contribute to neurofibroma formation and angiogenesis, while in UC, mast cell–nerve interactions in the gut perpetuate inflammation (11). Skin and intestinal biopsies in this case showed mast cell infiltration, supporting their putative role in the comorbidity. Previous studies also suggest mast cell dysregulation as a common pathway (9–11, 14). A 2014 study (11) reported increased c-kit/CD117^+^ mast cells and neuron specific enolase/S100^+^ Schwann cells in colonic mucosa from a patient with UC and NF1, a finding absent in isolated UC or normal mucosa. Our case provides additional histological support for this hypothesis.

Recent advances suggest that NF1 and UC may share pathophysiological pathways. NF1 patients are prone to gastrointestinal stromal tumors, which can mimic UC symptoms such as bleeding or abdominal pain (9). Moreover, the neurofibroma microenvironment in NF1 and the inflammatory milieu in UC may share features such as cytokine dysregulation and immune cell infiltration. Dysregulation of the RAS pathway—central to NF1 tumorigenesis due to loss of neurofibromin (1, 2), also contributes to immune dysfunction in UC. NF1-related mast cell infiltration may further exacerbate intestinal inflammation, as observed in case biopsies. These overlapping mechanisms may explain the co-occurrence of both diseases.

Treatment of UC with NF1 requires an individualized approach that considers their interaction. Conventional UC therapies such as corticosteroids or immunosuppressants may be ineffective in some patients. The present case, for instance, had steroid-dependent UC but responded markedly to vedolizumab combined with SFEC, underscoring the need for tailored treatment. Management of NF1 remains primarily symptomatic, including surgical excision of symptomatic neurofibromas. Close monitoring and timely adjustment of treatment strategies are essential. Most reported cases used corticosteroids or 5-aminosalicylic acid (10, 12, 16); the favorable response to vedolizumab plus herbal medicine here may offer new options for treatment-resistant cases.

## Conclusion

This case report details an elderly male patient suffering from steroid-dependent UC and NF1. The patient achieved remission through a combination therapy with vedolizumab and SFEC. The clinical manifestations of NF1 in this patient were evident in various areas, including the skin, bones, gastrointestinal tract, and omentum majus. Notably, the intestinal ulcers and polypoid lesions may be associated with the presence of neurofibromas and exhibited signs of mast cell infiltration. This case underscores potential shared mechanisms between NF1 and UC, such as immune microenvironment interactions and the duality of the RAS signaling pathway. It emphasizes the necessity for a multidisciplinary approach in managing complex and rare diseases.

## Data Availability

The original contributions presented in this study are included in this article/Supplementary material, further inquiries can be directed to the corresponding author.

## Supplementary material

Supplementary Figure 1 Colonoscopy examination data of the patient’s subcutaneous nodules. (A) HE staining. (B) S100 immunohistochemical staining. (C) CD117 immunohistochemical staining.
