# Supplementary figures and images for: Coexistence of ulcerative colitis and neurofibromatosis type 1: a case report and literature review
Source: Front Med (Lausanne). 2025 Nov 19;12:1723026. doi: 10.3389/fmed.2025.1723026 (PMC12672877; doi:10.3389/fmed.2025.1723026)

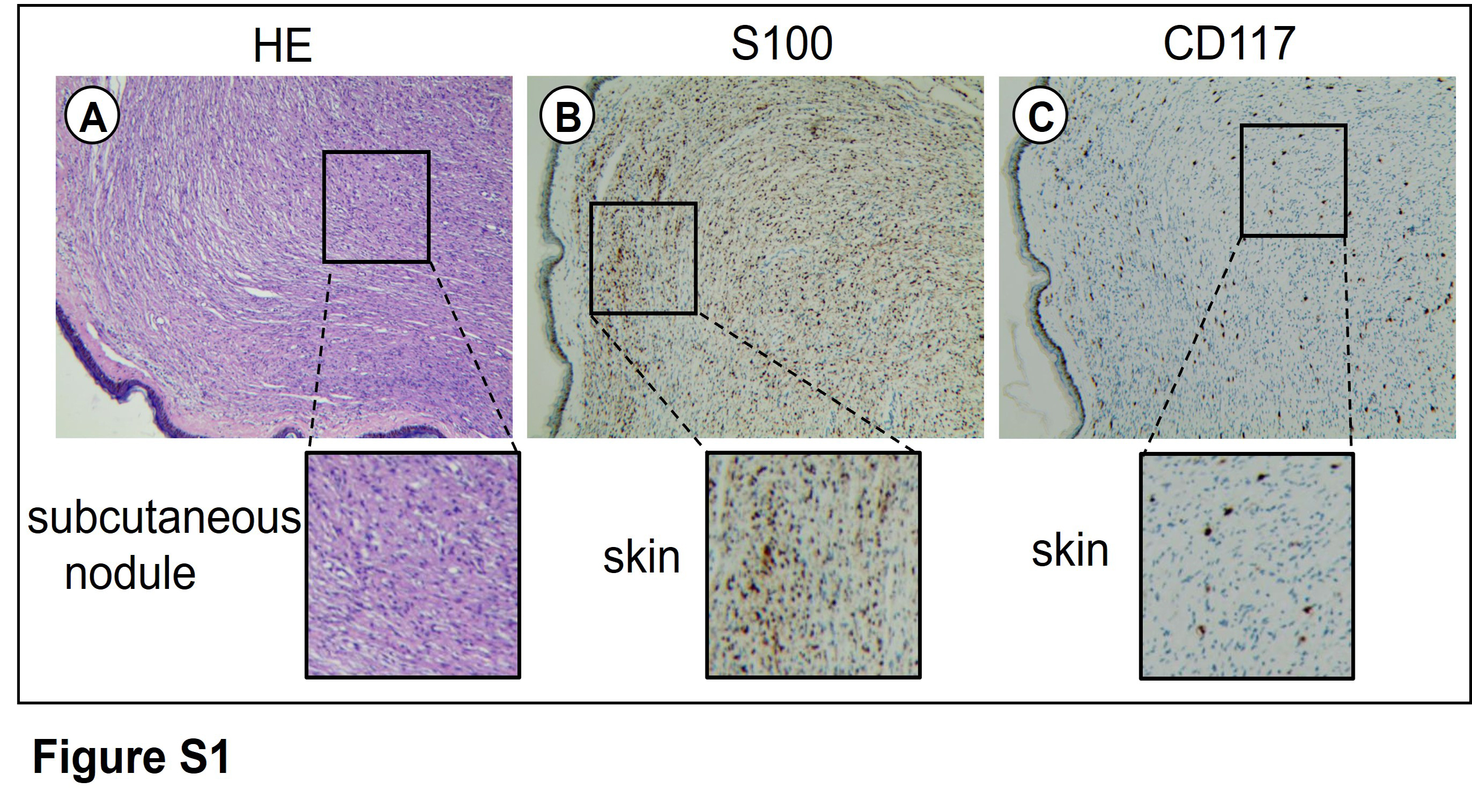

Supplement: Supplementary Figure 1 — Colonoscopy examination data of the patient’s subcutaneous nodules. (A) HE staining. (B) S100 immunohistochemical staining. (C) CD117 immunohistochemical staining. [file Image_1.jpeg]
